# Utilization of Medicinal Plants in Mental Disorders: Neuroplasticity and Neuroprotection in Biomodels

**DOI:** 10.3390/brainsci15040366

**Published:** 2025-03-31

**Authors:** Jose Luis Estela-Zape, Valeria Sanclemente-Cardoza, Lizeth Dayana Noreña-Buitrón, Leidy Tatiana Ordoñez-Mora

**Affiliations:** 1 Faculty of Health, Universidad Santiago de Cali, Cali 760035, Colombia; valeriasanclemente0@gmail.com (V.S.-C.); lizethdayana752@gmail.com (L.D.N.-B.); leidy.ordonez01@usc.edu.co (L.T.O.-M.); 2 Faculty of Health, Posgrado en Ciencias Biomédicas, Universidad del Valle, Cali 760043, Colombia

**Keywords:** medicinal plants, neuroplasticity, neurotransmitters, mental disorders, phytochemistry

## Abstract

Background/Objectives: Mental disorders such as anxiety, schizophrenia, and depression are linked to alterations in neuroplasticity and neuroprotection within the central nervous system. While conventional drugs are widely used, medicinal plants are emerging as a promising alternative due to their potential therapeutic effects on neuronal function. This study aimed to explore and analyze the impact of medicinal plants on neuroplasticity and neuroprotection in relation to mental disorders using biomodels. Methods: Data were collected from Scopus, Dimensions, and PubMed by using the search terms “Medicinal plants”, “Neuronal Plasticity”, and “Mental Disorder” in accordance with the guidelines of the PRISMA checklist. Results: A total of twenty-three relevant studies were selected to investigate the association between medicinal plants and mental disorders, focusing on factors such as administered doses and the modulation of neurotransmitters in the context of neuroplasticity and neuroprotection. This review highlights the complexity of study designs, target populations, and methodologies. Of the studies, 86% investigated depression, while 13% focused on anxiety. Regarding neurotransmitter modulation, 47% found that medicinal plants influenced serotonin levels, followed by 27% which found that they affected dopamine; according to the remaining studies, medicinal plants impacted norepinephrine, GABA, and acetylcholine. These findings emphasize the importance of precise dosing and neurotransmitter modulation, suggesting that targeted interactions with neural systems may help clarify the specific effects of these plants on mental health. Conclusions: Research on the effects of medicinal plants on psychiatric disorders in animal models suggests their potential to support neuroplasticity and neuroprotection. Positive impacts on mental health are indicated through the modulation of cytokines, neurotransmitters, and specific signaling pathways.

## 1. Introduction

The central nervous system (CNS), as the body’s control axis, governs essential functions, including movement, cognition, and emotional regulation [1]. Neuronal dynamics support neuroplasticity and neuroprotection processes, facilitating adaptation and recovery from internal and external damage [2,3]. These processes rely on neurophysiological mechanisms such as synaptogenesis, synaptic plasticity, and neurogenesis, which enable the formation of new connections, the adjustment of existing synaptic strengths, and neuronal genesis [4]. By preserving cellular and neuronal integrity, neuroprotection fosters an environment conducive to neuroplasticity, promoting optimal CNS function [5].

**Figure 1:**
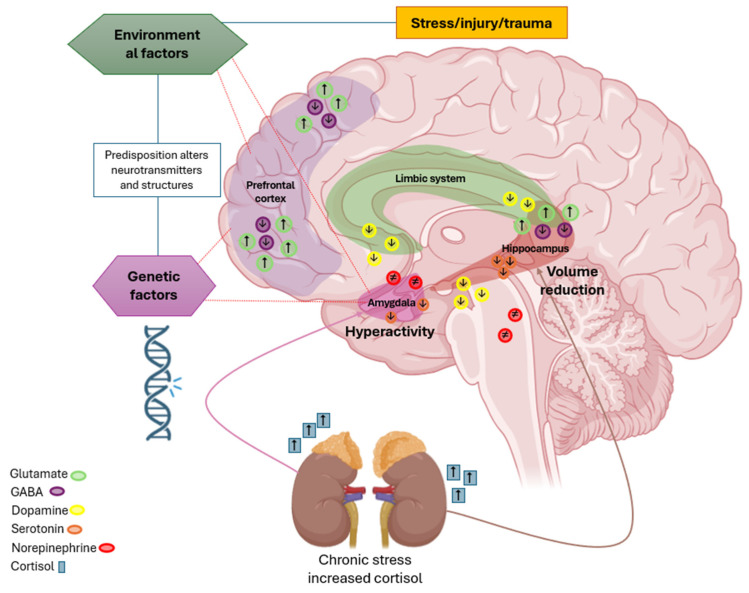
Pathophysiology of anxiety, depression, and stress. Note: Upward arrows imply increase, downward arrows decrease, sign (≠) inhibition of the neurotransmitter.

Mental disorders (MDs) in humans, such as anxiety, schizophrenia, and depression, are associated with altered neuroplasticity and neuroprotection mechanisms within the CNS, leading to morphophysiological changes in brain structures and affecting emotional and cognitive functions [6]. Studies indicate that anxiety disrupts synaptic plasticity, impairing adaptive responses to stress. Schizophrenia induces imbalances in synaptic plasticity and neuronal connectivity, impacting perception, cognition, and emotions [7,8]. Depression is linked to reduced neurogenesis in the hippocampus and prefrontal cortex, compromising mood regulation and emotional resilience (Figure 1) [9].

Key brain areas include the amygdala, which mediates responses to fear and anxiety; the hippocampus, whose atrophy is associated with depressive symptoms; and the prefrontal cortex, responsible for emotional regulation and decision making. Genetic and environmental factors influence the regulation of neurotransmitters such as serotonin, dopamine, norepinephrine, GABA, and glutamate. Decreased serotonin levels are linked to depressive symptoms, while reduced dopamine contributes to anhedonia and motivation deficits. The dysregulation of norepinephrine is associated with heightened anxiety, and decreased GABA levels result in increased neuronal excitability. Excess glutamate can lead to synaptic hyperactivity, also associated with anxiety. The activation of the hypothalamic–pituitary–adrenal (HPA) axis in response to chronic stress elevates cortisol levels, resulting in amygdala hyperactivity and reduced hippocampal volume, thereby exacerbating symptoms of anxiety, depression, and stress-related disorders.

Dysfunctions in neuronal dynamics signify brain function deterioration, impacting both mental health and global economic costs. According to the World Health Organization (WHO), the economic burden is estimated at approximately USD one trillion annually, with an alarming 13% increase in MDs [10,11,12].

Standard treatments for mental disorders include antidepressants, mood stabilizers, anxiolytics, and antipsychotics, which modulate neuronal activity and contribute to neuroprotection and neuroplasticity, potentially mitigating cognitive decline in neurodegenerative diseases [13,14]. Research shows that anti-inflammatory agents and antioxidants like vitamins C, E, and A act within the CNS to protect cells from oxidative stress and reduce inflammation, thereby supporting neuronal plasticity. In Parkinson’s disease, certain drugs act as metabolic precursors to dopamine, preventing the degradation of dopaminergic neurons due to mitochondrial dysfunction and apoptotic processes typical of neurodegenerative conditions. Additionally, antidepressants and anxiolytics, particularly neurotransmitter reuptake inhibitors, promote neurogenesis in regions such as the hippocampus, enhancing dendritic growth and neuronal connectivity.

Nevertheless, these therapies face limitations due to complex changes in neuronal dynamics, including receptor imbalances, altered neurotransmitter expression, and reduced neurogenesis. Such factors can lead to decreased efficacy, side effects, and drug resistance, highlighting the need for more effective and tolerable therapeutic alternatives [2].

Medicinal plants have been proposed as a promising alternative in the academic field because of their wide spectrum of therapeutic properties capable of influencing neuronal dynamics, whether in animal or human studies. These properties range from the activation of receptors and enzyme inhibition to the modulation of gene expression and the regulation of neurotransmitters, such as dopamine, serotonin, and gamma-aminobutyric acid (GABA), which contribute to cellular protection and exhibit antioxidant effects [15,16].

Research has shown that Lavandula angustifolia and *Valeriana jatamansi* can modulate the GABAergic system, addressing anxiety and insomnia [17,18]. In preclinical models, species like *Crocus sativus* [18], *Coffea arabica*, and *Bacopa monnieri* have demonstrated potential in regulating brain-derived neurotrophic factor (BDNF), a key factor in neurogenesis, synaptic plasticity, and neuronal survival, contributing to antidepressant effects during chronic stress [19,20,21].

Studies also suggest that *Crocus sativus*, *Coffea arabica*, and *Bacopa monnieri* exhibit neuroprotective properties, potentially counteracting β-amyloid neurotoxicity, oxidative stress, and glutamate excitotoxicity. Additionally, cannabidiol (CBD) may not only regulate BDNF but also mitigate the adverse effects of tetrahydrocannabinol (THC), supporting neuronal protection and synaptic remodeling [22]. *Rhodiola rosea* and *Ginkgo biloba* may reduce inflammation, protect neurons from oxidative damage, enhance synaptic plasticity, and reduce cell death, showing promise for managing depression and schizophrenia [14,23].

The effects of medicinal plants on mental disorders remain insufficiently understood, with inconclusive and heterogeneous findings. While some studies suggest therapeutic potential, significant gaps and inconsistencies persist regarding non-pharmacological treatments. This underscores the need for rigorous research on various plant species documented in scientific literature. Accordingly, a systematic review is proposed to evaluate and analyze the effects of medicinal plants on neuroplasticity and neuroprotection in mental disorders using biomodels. This review aims to consolidate current knowledge, deepening our understanding of the therapeutic properties of medicinal plants and their impact on complex neuronal dynamics associated with mental disorders in animals.

## 2. Materials and Methods

A systematic review of the literature was carried out according to the guidelines established by the PRISMA checklist [24] to ensure uniformity and quality in the presentation of the reports.

### 2.1. Search Strategy

#### 2.1.1. Source of Information

A search strategy was developed for SCOPUS, DIMENSIONS, and PUBMED, tailored to each database and incorporating combinations of medical subject headings and free-text terms related to mental disorders, medicinal plants, and study types. The targeted search included indexed terms and free text from sources such as abstracts and ongoing clinical trials. Articles were included in any language and without restriction on the publication date.

#### 2.1.2. Review Question

The research question was formulated and developed by using the PICO strategy: “What are the effects of medicinal plants on neuroplasticity and neuroprotection in mental disorders in biomodels?” The following elements were determined:

- Population: Biomodels used in the study of mental disorders.
- Intervention: Administration of medicinal plants.
- Comparison: Not applicable.
- Outcomes: Measures of neuroplasticity and neuroprotection.

#### 2.1.3. Search Terms

The search was carried out by using DeCS/MeSH terms, together with the logical operators “OR” and “AND”, with the purpose of constructing a search equation, i.e., (((((((Plants) OR (Medicinal plants)) OR (Phytochemicals)) OR (Traditional Medicine)) OR (Phytotherapy)) AND (Neuronal Plasticity)) AND (Neuroprotective Agents)) AND (Mental Disorder), directly related to the research goal. Appendix A shows the search strategies used in the different databases.

#### 2.1.4. Eligibility and Exclusion Criteria

Studies were included if they evaluated the effects of medicinal plants on mental disorders, specifically anxiety disorder, bipolar disorder, depression, stress, attention deficit hyperactivity disorder, and schizophrenia, considered as primary and secondary outcomes. Only randomized controlled trials (RCTs) and original research articles were included, with no publication date restrictions.

Exclusion criteria were set to omit studies focusing on Alzheimer’s disease, Parkinson’s disease, and Huntington’s disease, as these are classified as neurocognitive disorders in the Diagnostic and Statistical Manual of Mental Disorders (DSM-5) [25]. Additionally, we excluded studies not related to mental disorders, neuroplasticity, or neuroprotection; those involving combinations of plants, synthesized plant-derived compounds, extracts, or commercial medications; and those with incomplete data, reviews, or gray literature.

### 2.2. Study Selection

Studies were selected through a rigorous calibration procedure. Initially, three researchers (L.D.N.B., V.S.C., and L.T.O.M.) independently and blindly carried out the filtering process in various databases.

Each researcher prepared a list of studies that met the inclusion criteria after evaluating the title and abstract of the articles. In situations in which opposition arose among the three reviewers, a fourth reviewer (J.L.E.Z.), an expert on the subject, made the final decision regarding the inclusion of the articles. It is essential to note that the review process was blinded.

Eligibility criteria were applied during the full-text analysis phase in the final selection stage, and any disagreement among the authors regarding the appropriateness, eligibility, quality, or data obtained from the studies was resolved by consensus.

### 2.3. Quality Evaluation

The SYRCLE Risk of Bias (RoB) tool [26], adapted from the Cochrane RoB tool, was used to conduct a comprehensive methodological assessment of the animal intervention studies included in the analysis. This tool addresses bias factors specific to animal studies and contains ten items: six related to types of bias, one evaluating baseline characteristic similarity between experimental and control groups, one assessing the randomization of the housing conditions, one indicating whether disease induction occurred before or after randomization, and one evaluating blinding. Studies were rated “yes” (green) for low risk of bias, “no” (red) for high risk, and “unclear” (yellow) for uncertain risk. Only studies scoring six or higher were included.

## 3. Results

**Figure 2:**
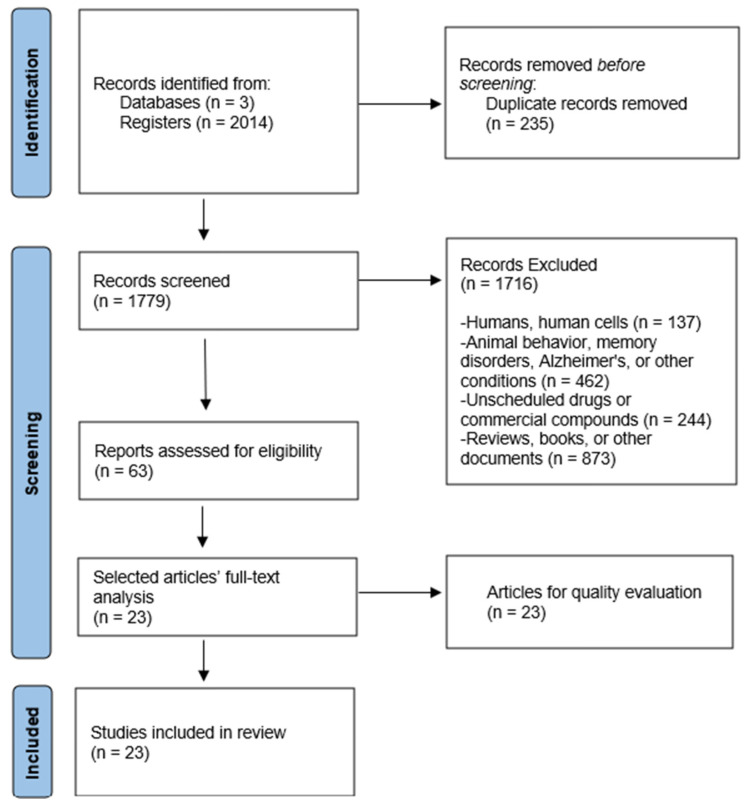
Flowchart of study selection process.

A total of 2014 records were initially identified through database analysis. After the removal of duplicates, a detailed review of 1779 articles was conducted, applying inclusion and exclusion criteria to finalize the selection. This process resulted in the identification of 63 relevant studies. Ultimately, 23 articles were included in this review, and their methodological quality was assessed by using the SYRCLE Risk of Bias (RoB) tool [26]. Figure 2 presents a graphical representation of the study selection process.

### 3.1. Methodological Quality

Out of the 23 studies evaluated, 65.2% (15 studies) obtained scores of 7, 8, and 9 when analyzed by using the RoB tool [26], which reflects a low risk of bias and high methodological quality. In contrast, only 8.69% (two studies) scored 4, suggesting a high risk of bias. The main deficiencies identified in these cases were inadequate randomization procedures, a lack of specification in concealment processes, and difficulties in blinding. A detailed methodological evaluation of each study is presented in Table 1.

### 3.2. Data Extraction and Synthesis

The study design, target population, and methodology differed among the studies, thus providing a comprehensive approach to analyzing the relationship between medicinal plants and mental disorders, as shown in Table 2. The considerable variability in the study design, populations studied, and methodologies used highlights the complexity inherent in the exploration of the association between medicinal plants and mental disorders.

As shown in Table 3, 86.6% of the studies included focused on depression, while 13.4% addressed anxiety. Regarding neurotransmitter effects, 47% (11 studies) of the medicinal plants studied affected serotonin, 27% (4 studies) influenced dopamine, and the remaining 26% impacted norepinephrine, GABA, and acetylcholine. The attention paid to dosage and neurotransmitter modulation highlights the necessity for precise administration and targeted interaction with neuronal systems to better understand the specific effects of these plants on the nervous system. These findings not only strengthen current knowledge but also provide a robust foundation for future research and clinical applications in psychiatric phytotherapy.

## 4. Discussion

The analysis conducted in this study suggests the importance of medicinal plants in addressing prevalent psychiatric disorders, including anxiety and depression, highlighting their potential therapeutic potential in the context of neuroplasticity and neuroprotection.

### 4.1. Phytomedicine Impact on Cytokines, Neurotransmitters, and Neuroplasticity

All gathered studies focused on the effects of phytomedicine in animal models, mostly using adult male mice as a standard population. It should be noted, however, that several studies chose to include female mice because of the greater susceptibility to depression [35,38]. These analyses followed a design that combined in vivo and in vitro approaches, with the specific addition of in silico models [36,40].

**Figure 3:**
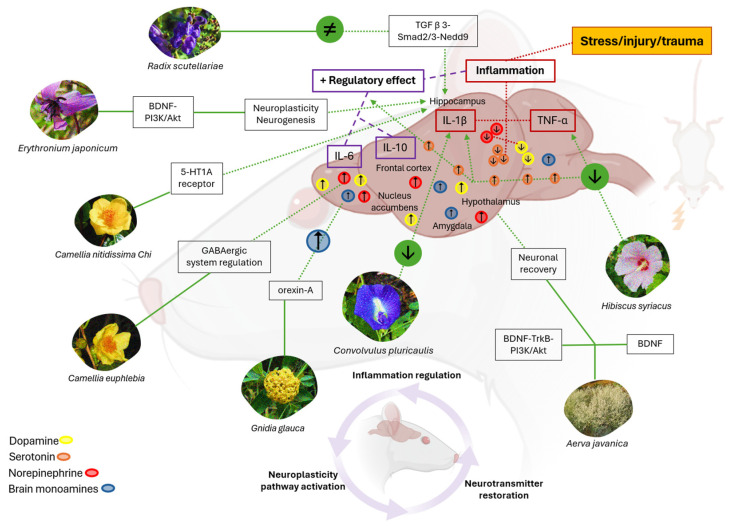
Regulation of cytokines, neurotransmitters, and neuroplasticity. Note: Ascending arrows imply increase, descending arrows decrease, sign (≠) inhibition of the neurotransmitter.

Environmental and psychosocial stressors were generated during the trials to induce chronic stress, which allowed us to obtain evidence of destabilization in the balance of cytokines (tumor necrosis factor alpha (TNF-α), interleukin-1 beta (IL-1β), interleukin-6 (IL-6), and interleukin-10 (IL-10)), signaling molecules in the immune response and neuronal communication (Figure 3).

Cytokine imbalance is associated with a reduction in brain amines, including serotonin, dopamine, and norepinephrine, which regulate emotional and cognitive functions. These biochemical changes show a complex interaction between prolonged stress and neurological homeostasis, which may contribute to the development of depression, brain inflammation, and neurotransmitter deregulation. These factors affect the neurochemical mechanisms and neuronal pathways involved in psychiatric disorders [27].

Stress increases inflammatory cytokines (TNF-α, IL-1β, and IL-6), which disrupt neurotransmitter levels (serotonin, dopamine, and norepinephrine), contributing to disorders such as anxiety, depression, and neuroinflammation. Plants like *Camellia euphlebia*, *Camellia nitidissima* Chi, *Gnidia glauca*, *Convolvulus pluricaulis*, *Hibiscus syriacus*, and *Erythronium japonicum* exert anti-inflammatory effects by regulating cytokine levels and restoring neurotransmitter balance, alleviating symptoms of anxiety and depression. Additionally, *Aerva javanica* and *Radix Scutellariae* activate neuroplasticity pathways, such as BDNF–PI3K/Akt, which promote neuronal growth and recovery in the hippocampus, mitigating the effects of chronic stress and enhancing overall cognitive function.

Standardized behavioral tests, such as the forced swim test (FST) and the tail suspension test (TST), are essential tools to determine the therapeutic effects of medicinal plants [31]. For example, He et al. (2015) [33] found that the aqueous extract of *Camellia euphlebia* has anxiolytic effects in the TST, thus alleviating the dysfunction of the GABAergic system and regulating neuronal excitation in mood disorders. Similarly, Tsoi et al. (2022) [37] reported that *Camellia nitidissima* Chi extract reduces stress and anxiety, thus regulating the hypothalamic–pituitary–adrenocortical (HPA) system and inhibiting chronic corticosterone (CORT) levels. This allows for the modulation of the serotonergic system and 5-HTR1A receptor expression in the hippocampus of mice.

Furthermore, Arika et al. (2019) [38] studied *Gnidia glauca* using the FST and TST and demonstrated the ability to modulate anxiety through the action of the neuropeptide orexin-A and the association between the increase in brain levels of mood-regulating monoamines. Lim et al. (2020) [41] also found that *Erythoronium japonicum* leaves activate the BDNF–PI3K/Akt pathway in the hippocampus of mice, involved in neuronal growth, survival, and plasticity. When activated by PI3K/Akt, BDNF appears to influence the regulation of the inflammatory response in the brain, thus exerting anti-inflammatory effects and improving depressive behavior in animal models.

The sucrose preference test (SPT) has been used to evaluate anhedonia, an important marker of depressive disorders in mice [39]. In the study conducted by Kim et al. (2018) [29], the use of *Hibiscus syriacus* L root reduced pro-inflammatory cytokines (IL-1β, IL-6, IL-8, and TNF-α), which correlated with an increase in the brain levels of serotonin. The findings indicate a potential neuroprotective and inflammation-regulating effect, which could positively impact emotional well-being. Arshad, H.M. et al. (2022) [36], however, demonstrated that the use of Aerva javanica leaves increased BDNF expression through the compound’s quercetin and kaempferol, which generated an antidepressant effect through the BDNF–TrkB–PI3K/Akt pathway.

Other studies [27,39] determined that *Convolvulus pluricaulis* extract markedly reduced IL-1β in the plasma of rats and decreased elevated levels of ALT liver enzymes. *Radix Scutellariae*, on the other hand, regulated neuroprotection in the hippocampus of mice, by inhibiting neuronal apoptosis through the TGF β 3–Smad2/3–Nedd9 signaling pathway. These mechanisms may be necessary to preserve neuronal integrity and reduce harmful processes that could contribute to neuropsychiatric diseases.

### 4.2. Role of Neurotransmitters in Neuroplasticity and Neuroprotection

**Figure 4:**
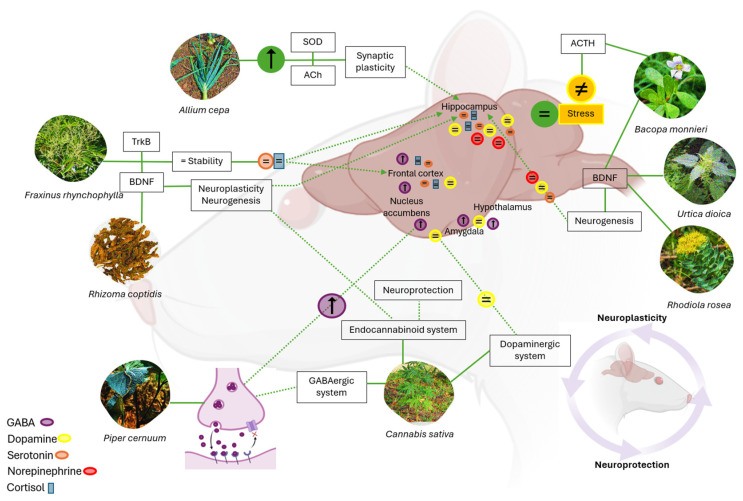
Neuroplasticity and neuroprotection effects of medicinal plants. Note: Ascending arrows imply increase, descending arrows decrease, sign (≠) inhibition, sign = neurotransmitter regulation.

The impact of medicinal plants on neuroplasticity and neuroprotection in mental disorders has been demonstrated in biomodels studies, which show that their bioactive compounds can modulate neurotransmitters, reduce oxidative stress, and promote neurogenesis [42,49] (Figure 4). Liu et al. (2023) [43] note that the hippocampus regulates neuroplasticity, and in depression, synapses in this region deteriorate, leading to the dysregulation of serotonin (5-HT) levels.

Neurotransmitters such as serotonin and norepinephrine play a key role in regulating mood in depression [48]. Additionally, depression is associated with decreased enzymatic antioxidant defenses and increased levels of reactive oxygen species [44]. Samad et al. (2018) [28] investigated the role of acetylcholine in the development of anxiety and depression. Their findings suggest that *Allium cepa* has antidepressant and anxiolytic effects by enhancing the activity of superoxide dismutase (SOD), an enzyme that reduces oxidative stress and cellular damage. Additionally, it increases acetylcholinesterase (AChE) activity, which modulates acetylcholine availability in neuronal synapses.

Medicinal plants such as Allium cepa, Fraxinus rhynchophylla, Bacopa monnieri, Urtica dioica, Rhizoma coptidis, Piper cernuum, Rhodiola rosea, and Cannabis sativa have beneficial effects on neuroplasticity and neuroprotection. Allium cepa increases superoxide dismutase (SOD) and acetylcholine, reducing oxidative stress. Fraxinus rhynchophylla normalizes serotonin and cortisol levels, promoting neurogenesis. Bacopa monnieri increases brain-derived neurotrophic factor (BDNF) and regulates adrenocorticotropic hormone (ACTH), supporting new-neuron formation. Urtica dioica modulates the hormonal axis and enhances neuronal regeneration. Rhizoma coptidis increases BDNF, alleviating stress. Piper cernuum enhances synaptic plasticity and modulates neuronal excitability by increasing GABA levels. Rhodiola rosea regulates key neurotransmitters and protects neurons from stress-induced damage. Cannabis sativa, through THC and CBD, influences synaptic plasticity, neurogenesis, and emotional regulation, supporting cognitive health.

Kim, R. et al. (2018) [30] observed that *Fraxinus rhynchophylla* Hance extract improves anxiety and depressive behavior. This effect can be attributed to the normalization of serotonin and cortisol levels, which affect BDNF and TrkB signaling in the hippocampus of mice. Furthermore, other studies [31,33,38] have determined that extracts from plants, including *Bacopa monnieri*, *Urtica dioica*, and *Rhizoma coptidis*, affect hormonal regulation and neurogenesis in the hippocampus of rats. The extracts appear to affect the production of adrenocorticotropic hormone (ACTH), related to stress, and the increase in BDNF, thus promoting the formation of new neurons. These components could help improve neuronal plasticity and protect brain cells.

Conversely, the study by Maia, M. et al. (2023) [35] examined the anxiolytic potential of *Piper cernuum* leaves by increasing GABA levels and improving the GABAergic system. The increase in GABA could be associated with the reduction in neuronal excitability and, potentially, could improve synaptic plasticity to mitigate these disorders, considering that GABA is the main inhibitory neurotransmitter in the brain and its alteration is related to anxiety and depression [40,41].

The potential of medicinal plants for neuroplasticity and neuroprotection in psychiatric disorders is an emerging research area. Current evidence, primarily from animal models, indicates that certain plant extracts can affect key neurochemical pathways, such as the BDNF–PI3K/Akt system and the GABAergic system, enhancing mood regulation and reducing brain inflammation. Notable examples include flavonoids from fruits and vegetables, cannabinoids from *Cannabis sativa*, and adaptogens like *Rhodiola rosea*, which demonstrate neuroprotective and neuroplastic properties by modulating cortical plasticity and optimizing neuronal synapses.

However, reliance on animal models limits the direct translation of these findings into human clinical practice. Variability in plant extracts and a lack of methodological standardization restrict clinical applicability. The diversity of specific compounds and mechanisms of action further complicates the generalization of results.

To advance this field, rigorous clinical studies are necessary to assess the efficacy and safety of medicinal plants in humans. Additionally, a multidisciplinary and integrative approach is crucial to developing precise and personalized phytotherapeutic treatments, tailored to the specific characteristics of each neuropsychiatric disorder and individual patient needs.

Despite these limitations, this study is notable for its methodological diversity, encompassing molecular analyses and standardized behavioral tests. The integration of vivo, in vitro, and in silico models strengthens the validity and comprehensiveness of the results, providing a solid foundation for future research and therapeutic advancements in phytotherapy with promising prospects for neuropsychiatric disorder treatment.

## 5. Conclusions

The investigation of molecular mechanisms in medicinal plants using animal models has identified critical pathways that enhance neuroplasticity and provide neuroprotection in psychiatric disorders. Specifically, the studies demonstrate how these plants modulate cytokine expression, regulate neurotransmitter systems, and activate neuroprotective signaling cascades. These findings highlight the therapeutic potential of specific medicinal plants and emphasize the need for focused research to identify the most promising candidates for clinical application. Advanced research could lead to novel treatments for neuropsychiatric disorders, significantly contributing to psychopharmacology and improving mental health outcomes.

## Figures and Tables

**Table 1:** Risk of bias (RoB).

	1	2	3	4	5	6	7	8	9	10	
Gupta, G. et al. (2019) [27]	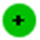	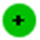	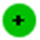	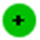	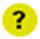	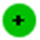	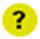	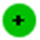	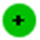	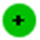	8
Samad, N. et al. (2017) [28]	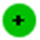	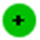	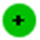	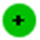	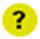	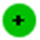	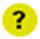	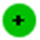	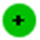	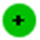	8
Kim, Y. et al. (2018) [29]	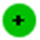	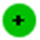	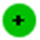	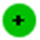	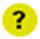	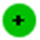	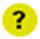	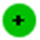	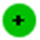	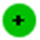	8
Kim, Y. et al. (2018) [30]	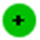	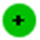	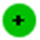	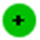	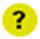	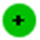	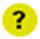	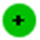	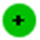	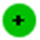	8
Kumar, S. et al. (2016) [31]	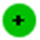	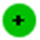	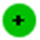	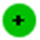	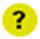	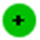	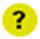	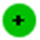	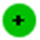	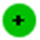	8
Chen, W. et al. (2016) [32]	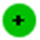	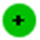	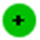	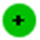	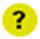	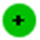	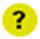	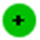	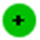	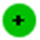	8
He, D. et al. (2015) [33]	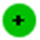	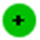	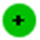	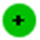	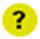	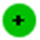	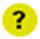	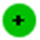	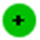	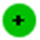	8
Patel, S. et al. (2014) [34]	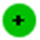	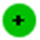	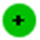	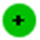	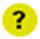	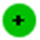	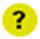	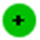	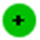	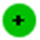	8
Maia, M. et al. (2023) [35]	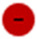	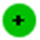	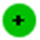	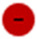	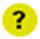	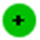	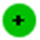	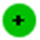	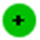	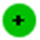	7
Arshad, H. et al. (2022) [36]	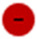	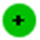	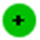	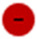	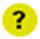	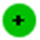	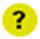	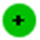	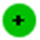	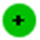	6
Tsoi, B. (2022) [37]	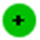	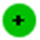	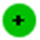	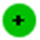	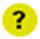	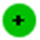	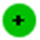	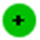	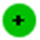	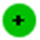	9
Arika, W. et al. (2019) [38]	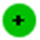	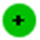	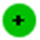	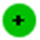	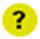	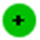	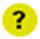	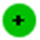	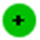	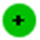	8
Zhao, F. et al. (2020) [39]	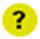	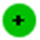	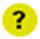	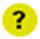	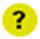	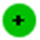	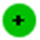	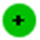	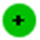	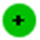	6
Qi, Y. et al. (2022) [40]	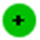	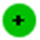	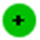	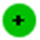	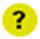	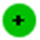	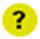	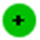	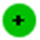	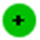	8
Lim, D. et al. (2020) [41]	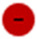	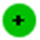	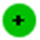	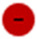	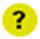	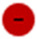	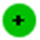	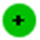	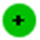	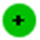	6
Yu, H. et al. (2022) [42]	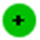	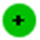	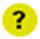	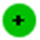	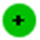	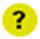	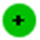	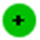	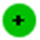	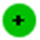	8
Liu, E.Y. et al. (2023) [43]	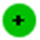	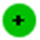	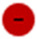	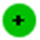	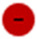	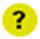	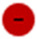	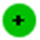	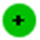	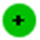	6
Ghazizadeh, J. et al. (2020) [44]	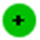	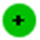	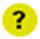	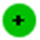	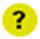	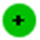	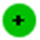	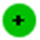	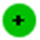	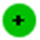	8
Zhao, F. et al. (2020) [39]	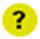	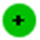	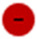	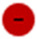	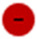	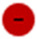	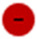	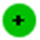	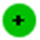	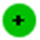	4
Guo, M. et al. (2024) [45]	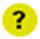	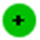	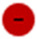	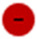	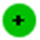	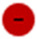	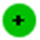	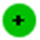	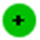	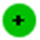	6
Fernandes, L.M. et al. (2024) [46]	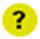	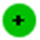	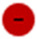	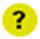	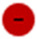	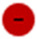	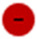	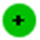	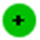	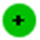	4
Tao, X. et al. (2024) [47]	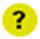	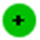	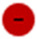	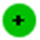	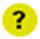	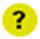	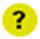	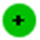	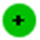	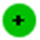	6
Li, J. et al. (2023) [48]	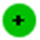	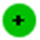	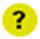	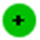	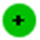	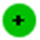	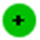	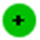	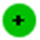	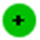	9

Green: Low risk, Yellow: Some concerns, Red: High risk.

**Table 2:** Design of studies found.

Author and Year	Plants	Family	Objective	Sample Size	Study Design	Control Group	Instruments	Results
Gupta, G. et al., 2019 [27]	*Convolvulus pluricaulis*	Convolvulaceae	Effects on neuroinflammation and monoamines in depression	36 male Wistar rats	In vivo, in vitro	6 groups: no stress + control, CUMS + control, 3 CPE groups, and CUMS + fluoxetine	Chromatography, acute toxicity, and blood and brain extraction	CPE reduced cytokines and enhanced neurotransmitter levels
Samad, N. et al., 2017 [28]	*Allium cepa*	Amarilidáceas	Impact on biochemical and behavioral changes	24 male albino Wistar rats	In vivo, in vitro	Onion extract vs. control	Brain tissue extraction	Improved anxiety, depression, and memory
Kim, Y. et al., 2018 [29]	*Hibiscus syriacus*	Malvaceae	Effects on depressive behaviors and neurotrophic factors	36 male C57/BL6 mice	In vivo, in vitro	6 groups: saline and stress + treatments	Cell culture and neuroblastoma cells	Reduced corticosterone levels
Kim, Y. et al., 2018 [30]	*Fraxinus rhynchophylla*	Oleaceae	Prevention of depressive behavior post-stress	Male C57BL/6 mice	In vivo, in vitro	PBS controls and FX treatments	Brain tissue samples	FX reduced depressive behaviors via serotonin modulation
Kumar, S. et al., 2016 [31]	*Bacopa monnieri*	Plantaginaceae	Neuroprotective effects on stress-induced depression	32 male Sprague-Dawley rats	In vivo and in vitro	4 groups: stress, BME, and IMI treatments	Biochemical assays and brain sectioning	BME reversed depressive effects by enhancing antioxidant levels
Chen, W. et al., 2016 [32]	*Gastrodia elata*	Orchidaceae	Antidepressant compounds and neurogenesis	40 male Sprague-Dawley rats	In vivo and in vitro	4 groups: WGE, GAS, and HBA treatments	HPLC-UV	WGE modulated monoamine metabolism
He, D. et al., 2015 [33]	*Camellia euphlebia*	Theaceae	Anxiolytic and antidepressant activities	30 male Kunming mice	In vivo and in vitro	5 groups: NaCl, diazepam, fluoxetine, and CEE	Brain homogenization	Increased neurotransmitters and dopamine release
Patel, S. et al., 2014 [34]	*Urtica dioica*	Urticaceae	Effects on diabetes-induced cognitive impairment	Adult Swiss albino mice	In vivo and in vitro	5 groups: dexamethasone and UD treatments	HPLC-UV	Reversed depressive behaviors by reducing oxidative stress
Maia, M. et al., 2023 [35]	*Piper cernuum*	Piperaceae	Neuropharmacological effects	Female Swiss mice	In vivo and in vitro	GABA estimation via spectrophotometry	GABA levels	Exhibited antidepressant and anxiolytic properties
Arshad, H. et al., 2022 [36]	*Aer Aerva javanica*	Amaranthaceae	Pharmacological activities in LPS-induced depression	60 male Swiss albino mice	In vivo, in vitro, and in silico	6 groups: saline, imipramine, and Aj Cr treatments	Molecular docking	Aj Cr showed antidepressant effects
Tsoi, B., 2022 [37]	*Camellia nitidissima*	Theaceae	Hippocampal neurogenesis and corticosterone-induced depression	72 male C57BL/6 N mice	In vivo and in vitro	6 groups: control, CORT, and CNC treatments	Plasma analysis and hippocampal neuron culture	CNC improved behavior through Akt/GSK3β/CREB signaling
Arika, W. et al., 2019 [38]	*Gnidia glauca*	Thymelaeaceae	Effects on locomotor and anxiety-like behaviors	30 female rats	In vivo	6 groups: diet + control and treatments	GC-MS	Increased locomotor and exploratory behavior
Zhao, F. et al., 2020 [39]	*Radix Scutellariae*	Lamiaceae	Antidepressant effects in CUMS model	50 male adult ICR mice	In vivo and in vitro	CUMS, fluoxetine, and RS treatments	Hippocampus extraction	Improved behaviors via TGF β pathway
Qi, Y. et al., 2022 [40]	*Coptis chinensis*	Ranunculaceae	Therapeutic mechanism in severe mental disorders	60 male SPF C57BL/6 mice	In vivo, in vitro, and in silico	6 groups: DZP and RC treatments	Blood and brain tissue extraction and molecular docking	Demonstrated anxiolytic effects
Lim, D. et al., 2020 [41]	*Erythronium japonicum*	Liliáceas	Anti-inflammatory effects in LPS-induced depression	50 male ICR mice	In vivo and in vitro	5 groups: sham, control, and treatments	Hippocampus extraction dose of EJE	Reduced neuroinflammation and depressive behaviors
Yu H; et al., 2022 [42]	*Diospyros kaki Thunb*	Ebenaceae	Activity on neurotransmitters in depression	CD-1 male mice	In vivo and in vitro	4 groups: low or high doses of PLE or fluoxetine	Golgi staining and immunofluorescence	It relieved depressive behaviors by inhibiting serotonin reuptake
Liu E; et al., 2023 [43]	*Uncaria rhynchophylla*	Rubiáceas	Antidepressant effects of RH	C57BL/6 male mice	In vivo and in vitro	6 groups with different doses of RH or fluoxetine	Western blot test	Increased 5-HT levels in the cortex and hippocampus
Ghazizadeh J et al., 2020 [44]	*Melissa officinalis*	Lamiaceae	Antidepressant effects of MO	60 male albino BALB/c mice	In vivo and in vitro	5 randomized groups with different stress techniques	Homogenization and TBAR assay	It attenuated stress-induced anxious and depressive behaviors
Zhao F. et al., 2020 [39]	*Radix Scutellariae*	Lamiaceae	Antidepressant effects and action on the TGF β signaling pathway	Adult male ICR mice	In vivo	2 groups	Immunohistochemistry and Nissl staining	Reversed the decrease in TGF β 3 protein
Guo, M; et al., 2024 [45]	*Gynostemma pentaphyllum*	Cucurbitaceae. G. pentaphyllum	Neuroprotective effects of Gyp on anxiety and depression	Mice	In vivo	Gyp and fluoxetine hydrochloride	Ultrasonic sonication and resin chromatography	Improved anxiety and depression
Fernandes, LM; et al., 2024 [46]	*Hybanthus enneaspermus*	Violaceae	Anxiolytic activity of ethanolic extract of Hybanthus enneaspermus	Mice	In vivo and in silico	*Hybanthus enneaspermus and diazepam hydrochloride*	Extraction, fractionation, and biofraction	Significantly mitigated anxiety
Tao, X; et al., 2024 [47]	*Ácido cajaninstilbeno*	Fabaceae	Effects of CSA on depressive behavior	Male C57BL/6 J and BALB/ mice	In vivo and in vitro	*2 groups: CSA*	Molecular analysis	It exerted antidepressant effects
Li, J; et al., 2023 [48]	*Sophora alopecuroides L.*	Fabáceas	Ameliorative effect of Sophora alopecuroides L. on depressive behavior	Mice	In vivo and in vitro	*ALK from Sophora alopecuroides L.*	Molecular biology and incubation	It showed antidepressant effects

CPE: *Convolvulus pluricaulis* extract; BDNF: brain-derived neurotrophic factor; ACTH: adrenocorticotropic hormone; CREB: cAMP response element-binding protein; GAS: *Gastrodia elata* stem extract; HBA: herbaceous extract from *Gastrodia elata*; 5-HT: serotonin; DA: dopamine; GABA: gamma-aminobutyric acid; FGVβ3: transforming growth factor beta 3; Nedd9: neural precursor cell expressed developmentally down-regulated 9; PI3K: phosphoinositide 3-kinase; Akt: protein kinase B; TGF β 3: transforming growth factor beta 3; SMAD2/3: SMAD family member 2/3; PFC: prefrontal cortex; IL-1β: interleukin-1 beta; NLRP3: NOD-like receptor family pyrin domain containing 3; ASC; apoptosis-associated speck-like protein containing a CARD; TLR4: Toll-like receptor 4; NF-κB: nuclear factor kappa-light-chain-enhancer of activated B cells; CSA: *Cajanus cajan* extract; ALKs: alkaloids.

**Table 3:** Influence on mental illnesses, doses, neurotransmitters, and nervous system.

Author and Year	Plants/Segment	Mental Disorders	Administration Duration and Dosage	Neurotransmitters	Effects on the Nervous System
Gupta, G. et al., 2019 [27]	*Convolvulus pluricaulis* (dried leaves)	Depression	50–100 mg/kg CPE or 10 mg/kg fluoxetine, once daily for 7 days	Serotonin and norepinephrine	Restored serotonin and norepinephrine levels in the hippocampus and prefrontal cortex
Samad, N. et al., 2017 [28]	*Allium cepa* (stem)	Anxiety and depression	200 mg/kg/day for 14 days	Acetylcholine	Increased brain acetylcholine, enhancing memory processes through neuroplasticity
Kim, Y. et al., 2018 [29]	*Hibiscus syriacus* (root)	Depression and stress	200 mg/kg for 22 days	Serotonin	Reduced depressive behavior via CREB/BDNF signaling, enhancing cognitive function
Kim, Y. et al., 2018 [30]	*Fraxinus rhynchophyl* (stem)	Depression	100–400 mg/kg for 2 weeks	Serotonin	Increased serotonin, decreased cortisol, and elevated BDNF in the hippocampus
Kumar, S. et al., 2016 [31]	*Bacopa monnieri* (leaves)	Depression	80 mg/kg	BDNF	Improved behavioral anomalies and increased ACTH, corticosterone, BDNF, and hippocampal neurogenesis
Chen, W. et al., 2016 [32]	*Gastrodia elata* (stem)	Depression	500 mg/kg WGE, 100 mg/kg GAS, and HBA for 2 weeks	Serotonin and monoamines	Decreased monoamine turnover and influenced the dopaminergic system
El, D. et al., 2015 [33]	*Camellia euphlebia* (leaves)	Anxiety and depression	100–400 mg/kg/day for 7 days	GABA, norepinephrine, and dopamine	Increased 5-HT and DA levels, providing anxiolytic and antidepressant effects
Patel, S. et al., 2014 [34]	*Urtica dioica* (leaves)	Depression	50–100 mg/kg/day	Acetylcholine	Modulated acetylcholine release, improving memory and depressive symptoms
Maia, M. et al., 2023 [35]	*Piper cernuum* (leaves)	Depression and anxiety	50–150 mg/kg for 15 days	GABA and serotonin	Increased GABA levels, optimizing neurotransmission
Arshad, H. et al., 2022 [36]	*Aerva javanic* (leaves)	Depression	100–500 mg/kg for 14 days	Norepinephrine, dopamine, catecholamines, and BDNF	Normalized BDNF levels, reduced oxidative stress, and mitigated depressive behavior
Tsoi, B; 2022 [37]	*Camellia nitidissima* (dried leaves)	Depression and anxiety	10–50 mg/kg for 40 days	Serotonin	Increased serotonin levels and promoted neurogenesis
Arika, W. et al., 2019 [38]	*Gnidia glauca* (fresh leaves)	Anxiety	200–300 mg/kg for 12 weeks	GABA and dopamine	Anxiolytic effects through dopamine release and GABAergic activation
Zhao, F. et al., 2020 [39]	*Radix Scutellariae* (dried leaves)	Depression	1.5 g/kg for 4 weeks	FGVβ3 and Nedd9	Modulated neuroprotection, anxiolytic effects, and TGF β 3–Smad2/3–Nedd9 pathway
Qi, Y. et al., 2022 [40]	*Coptis chinensis* (fresh leaves)	Anxiety	146–584 mg/kg/day for 6 days	Dopamine and serotonin	Provided neuroprotection by regulating inflammatory factors
Lim, D. et al., 2020 [41]	*Erythronium japonicum* (leaves)	Depression	100–300 mg/kg for 7 days	BDNF	Reduced inflammatory cytokines and improved depressive behavior by activating BDNF–PI3K/Akt pathway
Yu, H. et al., 2022 [42]	*Diospyros kaki Thunb* (leaves)	Depression	30–60 mg/kg or fluoxetine 10.0 mg/kg for 10 days	Serotonin	Inhibits 5HT reuptake and regulates the BDNF signaling pathway in the cortex
Liu, E. et al., 2023 [43]	*Uncaria rhynchophylla* (leaves)	Depression	RH at 25 mg/kg or fluoxetine 10 mg/kg for 28 days	Serotonin	Significantly increased 5-HT levels in the cortex
Ghazizadeh, J; et al., 2020 [44]	*Melissa officinalis* (leaves)	Depression and anxiety	MO at 50, 75, and 150 mg kg, for 14 days	Serotonin	Anti-inflammatory, antimicrobial, antioxidant, sedative, and neuroprotective effects
Zhao, F. et al., 2020 [39]	*Radix Scutellariae* (root)	Depression	RS at 0.75 g/kg and fluoxetine at 1.5 g/kg for 4 weeks	Serotonin and GABA	Mediated the TGF β 3–Smad2/3–Nedd9 signaling pathway, potential mechanism of the neuroprotective effect
Guo, M. et al., 2024 [45]	*Gynostemma pentaphyllum* (leaves)	Anxiety and depression	Gyp at 50, 100, or 200 mg/kg with fluoxetine hydrochloride	NLRP3/Caspase-1/ASC in PFC	Optimization in cytokine expression in the hippocampus and PFC, with IL-1β showing the most pronounced regulation
Fernandes, LM. et al., 2024 [46]	*Hybanthus enneaspermus* (leaves)	Anxiety	400 mg/kg Hybanthus enneaspermus	GABA, 5-HT, NA, and DA	Improved GABA levels, attenuated glutamate, and enhanced levels of NA, 5-HT, DA, and antioxidant enzymes
Tao, X. et al., 2024 [47]	Ácid cajaninstilbeno: *Cajanus cajan* (legume)	Depression	Group 1: CSA (7.5, 15, and 30 mg/kg) Group 2: CSA (7.5–30 mg/kg)	TLR4/NF-κB	It counteracted the activation of the TLR4/NF-κB pathway and the reduction in autophagy levels
Li, J. et al., 2023 [48]	*Sophora alopecuroides L.* (leaves)	Depression	ALK from Sophora alopecuroides L.	BDNF–AKT–mTOR	Antidepressant effect of ALKs from Sophora alopecuroides L. based on the BDNF–AKT–mTOR signaling pathway of the prefrontal cortex

CPE: *Convolvulus pluricaulis* extract; 5-HT: serotonin; DA: dopamine; GABA: gamma-aminobutyric acid; FGVβ3: transforming growth factor beta 3; Nedd9: neural precursor cell expressed developmentally down-regulated 9; BDNF: brain-derived neurotrophic factor; ACTH: adrenocorticotropic hormone; CREB: cAMP response element-binding protein; GAS: gastrodin; HBA: hesperidin; GABA: gamma-aminobutyric acid; NA: norepinephrine; CPE: Convolvulus pluricaulis extract; UD: *Urtica dioica* extract; HEPC: *Piper cernuum* hydroalcoholic extract; Aj Cr: *Aerva javanica* crude leaf extract; CNC: *Camellia nitidissima* extract; DCM: *Gnidia glauca* dichloromethane extract; RS: *Radix Scutellariae* extract; DZP: diazepam; RC: reference control; EJE: *Erythronium japonicum* extract; LPS: lipopolysaccharide; HPLC: high-performance liquid chromatography; PBS: phosphate-buffered saline; SK-N-SH: human neuroblastoma cells; CORT: corticosterone; RU486: mifepristone; PLE: Caqui leaves; RH: *Uncaria rhynchophylla*; MO: Melissa officinalis; TBAR: Thiobarbituric Acid Reactive Substances Assay; TLR4/NF-κB: Toll-like receptor 4/nuclear factor kappa-light-chain-enhancer of activated B cells; Gyp: gypenosides; CSA: cajaninstilbene acid; ALKs: alkaloids.

## Abbreviations

The following abbreviations are used in this manuscript:

CNS	central nervous system
MDs	mental disorders
HPA	hypothalamic–pituitary–adrenal
WHO	World Health Organization
GABA	gamma-aminobutyric acid
CBD	cannabidiol
BDNF	brain-derived neurotrophic factor
THC	tetrahydrocannabinol
TNF-α	tumor necrosis factor alpha
IL-1β	interleukin-1 beta
IL-6	interleukin-6
IL-10	interleukin-10
AChE	acetylcholinesterase

## Appendix A

**Table A1:** Search strategies for different databases.

Database	Search Date	Search Equation	Articles Found
Scopus	1 October 2025	(((((((Plants) OR (Medicinal plants)) OR (Phytochemicals)) OR (Traditional Medicine)) OR (Phytotherapy)) AND (Neuronal Plasticity)) AND (Neuroprotective Agents)) AND (Mental Disorder)	1950
PubMed	1 November 2025	(((((((Plants) OR (Medicinal plants)) OR (Phytochemicals)) OR (Traditional Medicine)) OR (Phytotherapy)) AND (Neuronal Plasticity)) AND (Neuroprotective Agents)) AND (Mental Disorder)	46
Dimensions	1 May 2025	(((((((Plants) OR (Medicinal plants)) OR (Phytochemicals)) OR (Traditional Medicine)) OR (Phytotherapy)) AND (Neuronal Plasticity)) AND (Neuroprotective Agents)) AND (Mental Disorder)	18
